# Chikungunya Myocarditis Mimicking Acute Coronary Syndrome in an Elderly Patient: A Case Report

**DOI:** 10.7759/cureus.98346

**Published:** 2025-12-02

**Authors:** Tharindu Gabadage

**Affiliations:** 1 Internal Medicine, Sri Jayawardenapura General Hospital, Colombo, LKA; 2 Internal Medicine, Postgraduate Institute of Medicine, Colombo, LKA

**Keywords:** cardiac complications, cardiac mri, chikungunya, heart failure, myocarditis

## Abstract

Chikungunya is a mosquito-borne viral infection. It causes mild fever, arthralgia, and rash in the majority of patients. Rarely, it can lead to serious cardiac complications such as myocarditis, primarily seen in patients with comorbidities. We present a 78-year-old elderly male with diabetes, hypertension, and dyslipidemia who presented with acute heart failure following a febrile illness. Initially, he was managed as an acute coronary syndrome. However, positive chikungunya IgM antibodies and a cardiac MRI demonstrating mid-wall and subepicardial late gadolinium enhancement confirmed chikungunya myocarditis. Chikungunya myocarditis is rare but can be seen in patients with comorbidities. Clinicians should maintain a high index of suspicion to diagnose myocarditis in patients with fever and cardiac symptoms. Early evaluation with imaging and serology will enable early diagnosis and help prevent adverse outcomes.

## Introduction

Chikungunya virus is an RNA virus belonging to the *Togaviridae* family and the *Alphavirus* genus. *Aedes aegypti* and *Aedes albopictus* mosquitoes transmit it. Chikungunya is widely distributed across tropical and subtropical regions [1].

Most patients experience mild illness with fever, arthralgia, and rash, while approximately 15% of patients remain asymptomatic. In most cases, complete recovery occurs without complications. However, in vulnerable groups such as infants and the elderly, chikungunya infection can lead to severe or life-threatening disease [2].

A systematic review on cardiovascular manifestations of chikungunya infection reported that 54.2% of affected patients showed some degree of cardiac involvement. Another meta-analysis of arboviral infections reported a mean incidence of cardiac events of approximately 33% among patients with chikungunya [3].

A broad spectrum of cardiac manifestations has been seen, including sinus tachycardia, arrhythmias, myocarditis, and even cardiac arrest [1]. Among these complications, myocarditis is rare, and its estimated prevalence is 2.38% [3].

Here, we present the case of a 78-year-old male with diabetes, hypertension, and dyslipidemia who developed acute decompensated heart failure secondary to myocarditis following chikungunya infection. This case highlights chikungunya myocarditis as a potential cause of acute heart failure, particularly during arboviral outbreaks.

## Case presentation

A 78-year-old male with a background history of diabetes mellitus, hypertension, and dyslipidemia presented with generalized body weakness for 10 days and shortness of breath for two days. He had been well for 10 days prior to admission. He then developed a high-grade fever with a headache lasting for three days, followed by arthralgia, myalgia, and loss of appetite. He also complained of orthopnea, paroxysmal nocturnal dyspnea, and bilateral lower limb swelling but denied chest pain, cough, cold, or sore throat. He had nausea without vomiting, and there was no abdominal pain or other gastrointestinal symptoms. His son and daughter had a history of fever with mild myalgia and arthralgia about two weeks earlier. It resolved within three to four days without medication, suggestive of a viral illness.

On examination, he was conscious and rational, with dyspnea at rest. His respiratory rate was 18 breaths per minute, and oxygen saturation was 88% on room air, improving to 99% with 8 L/min oxygen via face mask. Bilateral lower lobe crackles were noted. Blood pressure was 112/76 mmHg, pulse rate was 118 bpm, jugular venous pressure was elevated at 8 cm, and both S1 and S2 heart sounds were noted with no added heart sounds. Motor examination revealed strength of 4/5 in both upper limbs and 3+/5 in both lower limbs. The remainder of the neurological examination is normal. The abdomen was soft and non-tender.

**Figure 1:**
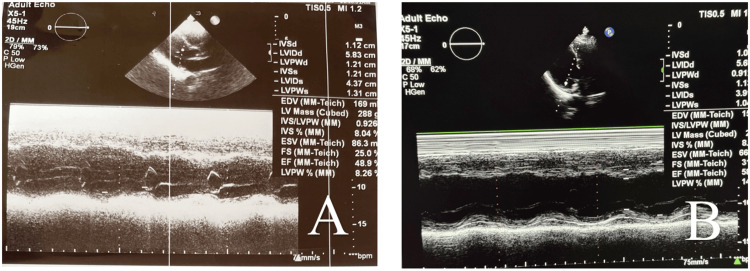
PLAX view of M-mode echocardiography (A) PLAX view on day 2 of admission showing a maximum ejection fraction of 48.9% after repeated measurements. (B) PLAX view at discharge showing improvement of ejection fraction to 58.3%, correlating with clinical recovery. PLAX: parasternal long-axis

**Figure 2:**
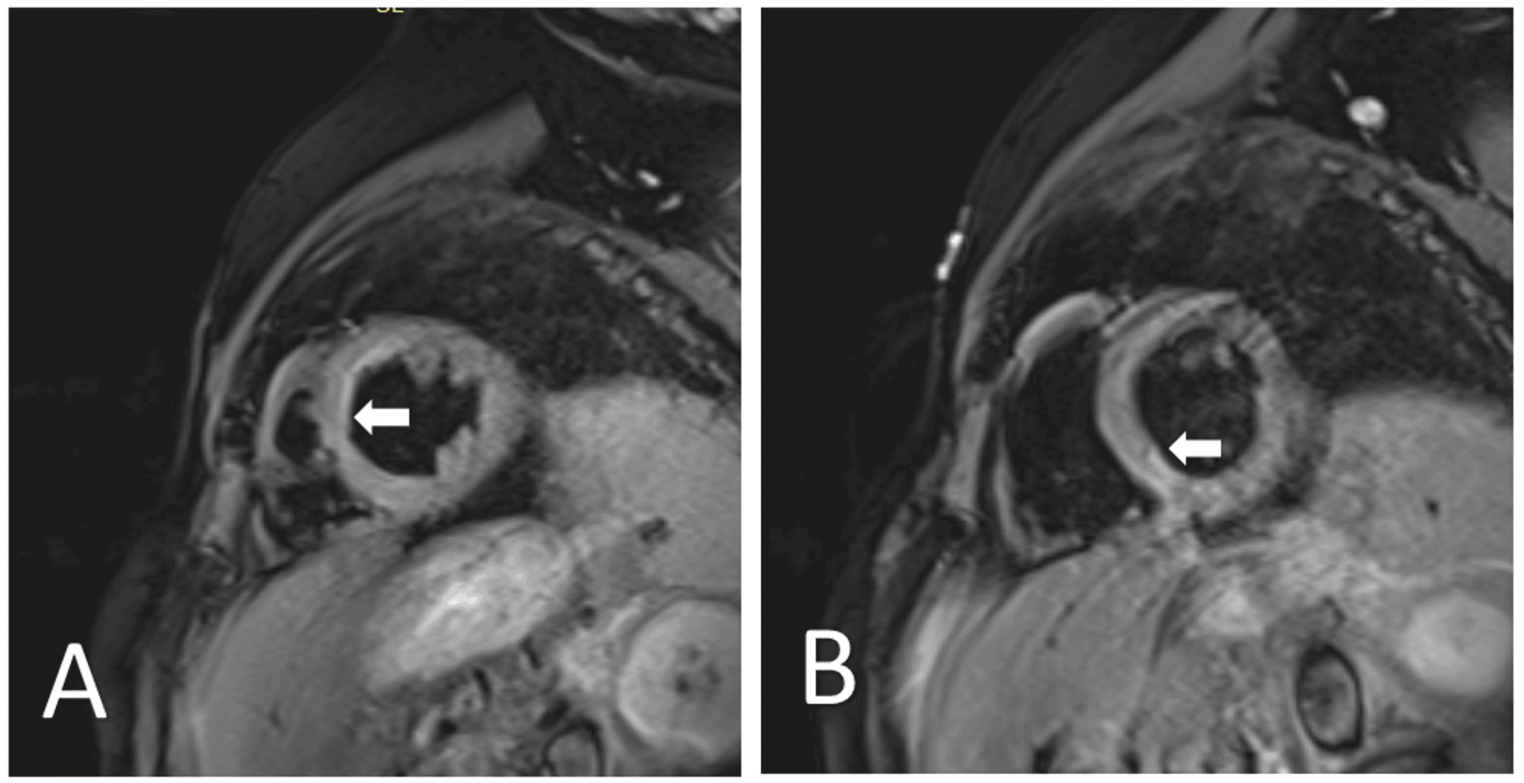
Cardiac MRI (short-axis view, late gadolinium enhancement sequence) The annotated areas in (A and B) demonstrate patchy areas of midwall to subepicardial enhancement predominantly involving the lateral wall of the left ventricle. There is no subendocardial or transmural enhancement in the coronary artery distribution. These findings are non-ischemic in pattern and are highly suggestive of myocarditis.

Bedside point-of-care ultrasonography revealed a left ventricular ejection fraction of 45% and a non-collapsing inferior vena cava and bilateral B-lines. Laboratory and imaging findings are summarized below (Tables 1-4, Figures 1-2).

**Table 1:** Summary of inflammatory markers WBC: white blood cells, Hb: hemoglobin, CRP: C-reactive protein

	Reference range	13/6/25	14/6/25	15/6/25	16/6/25	19/6/25	22/6/25
WBC	4.O-11.0 x 10^9^/L	4.1	5.1	5.7	7.5	8.93	7.12
Hb	11.0-16.0 g/dL	12.1	11.7	11.8	11.4	12.0	11.9
Platelet	150-450 x 10^9^/L	228	290	265	271	323	339
CRP	<6 mg/L	32	48	47	33	28	20

**Table 2:** Summary of biochemical investigations AST: aspartate transaminase, ALT: alanine transaminase, ALP: alkaline phosphatase, GGT: gamma-glutamyl transferase, BNP: brain natriuretic peptide, Na: sodium, K: potassium, ECG: electrocardiogram, HR: heart rate, CPK: creatine phosphokinase, LDH: lactate dehydrogenase

Investigation	Results	Reference range
AST	215	0-37 U/L
ALT	87	10-40 U/L
Total bilirubin	1.08	0.30-1.20 mg/dl
ALP	52	30-120 U/L
GGT	30	11-50 U/L
Total protein	5.9	6.0-8.0 g/dL
Albumin	2.9	3.7-5.0 g/dL
Globulin	3.0	2.0-4.0 g/dL
Serum creatinine	97	65-104 g/dL
Na	133	136-145 mmol/L
K	3.5	3.5-5.3 mmol/L
Troponin I	35.44	<0.034 ng/ml male
BNP	26000	<100 pg/mL
D-dimer	2341	<500 ng/ml
CPK	464	20-200U/L
Ferritin	397	12-263 g/L
LDH	680	105-248 U/L
Procalcitonin	0.29 ng/dL	0.05-0.5 ng/dL – low risk for progression to severe systemic sepsis

**Table 3:** Summary of cardiology, serological, microbiological, and radiological investigations DVT: deep vein thrombosis, LV: left ventricle, ED: end diastolic, ES: end systolic

Test	Results
ECG	Sinus tachycardia HR 136 bpm T inversion in lead II and aVf
Chikungunya IgM	Positive
Chikungunya IgG	Positive
COVID-19 antigen	Negative
Influenza A antigen	Negative
Influenza B antigen	Negative
Dengue IgM	Negative
Dengue IgG	Negative
DVT scan	Negative
Blood culture	No growth
Urine culture	No growth
Sputum culture	No growth
Cardiac MRI	Ejection fraction was 43%, with an LV ED volume of 129.7 mL, an LV ES volume of 73.1 mL, and an LV mass of 152.5 g. Patchy high–signal-intensity areas were observed in the mid-wall and subepicardial regions of the myocardium, particularly in the lateral and inferolateral walls of the left ventricle. These areas do not follow coronary artery distribution, a pattern more consistent with myocarditis than ischemic injury. No pericardial or pleural effusion was noted.
Coronary angiogram	Minor plaque disease

**Table 4:** 2D echocardiography summary An EF of 40-45% with mild anterior and anteroseptal hypokinesia was noted. By day 10, the EF had improved to 55-60% in parallel with clinical recovery. EF: ejection fraction

2D echocardiography	
Day 2 (14/6/2025)	Ejection fraction was 40-45%, with mild anterior and anteroseptal hypokinesia noted. Grade 1 diastolic dysfunction is present, with no evidence of pulmonary hypertension and no pericardial effusion.
Day 10 (22/6/2025)	Ejection fraction was 55-60%, with no regional wall motion abnormalities and good left ventricular function. No pericardial effusion was noted.

He was initially managed for non-ischemic myocardial infarction with acute heart failure using subcutaneous enoxaparin, dual antiplatelet therapy, intravenous frusemide, and continuous positive airway pressure. The diagnosis was supported by ECG changes in the inferior leads, elevated troponin I and BNP levels, and echocardiography findings, despite the absence of chest pain.

However, given the positive chikungunya IgM antibody, recent fever, ongoing arthralgia and myalgia with heart failure, chikungunya myocarditis was considered as a differential diagnosis. Following a multidisciplinary team discussion involving the cardiologist and radiologist, a cardiac MRI was performed, and it favored the diagnosis of myocarditis over acute coronary syndrome.

The patient showed clinical improvement, and he was weaned off oxygen and diuretics. A repeat echocardiography at discharge demonstrated improvement in left ventricular ejection fraction. A coronary angiogram was planned once the patient had recovered from the acute illness. It was performed four months after discharge and revealed only mild plaque disease, further supporting the diagnosis of myocarditis.

## Discussion

Chikungunya is an arboviral infection affecting over one million people worldwide annually. Its symptoms overlap with those of other endemic viral diseases such as dengue. This often leads to misdiagnosis and underestimation of its health burden [4]. Most infections are mild and self-limiting, usually resolving within two weeks, but some may progress to chronic or severe multi-organ involvement [2].

Cardiovascular complications are increasingly seen in chikungunya. A systematic review stated that cardiovascular involvement occurs in over half of patients with systemic manifestations, and a meta-analysis found that approximately one-third experience cardiac events [3,5]. Myocarditis is a serious complication resulting from direct viral invasion of cardiomyocytes and immune-mediated myocardial inflammation. It is rare, and the prevalence of viral myocarditis is 2.38% in one systematic review [3].

Preexisting conditions such as hypertension, diabetes mellitus, chronic kidney disease, and structural heart disease increase the risk of cardiac complications. A meta-analysis of 220,215 patients identified diabetes, hypertension, age ≥60 years, chronic kidney disease, male sex, and vomiting as significant predictors of mortality in chikungunya [6]. Our patient had multiple comorbidities from the above-identified risk factors, which may likely contribute to the development of myocarditis.

The patient presented with acute decompensated heart failure following a febrile illness. He had features compatible with acute coronary syndrome, including elevated cardiac enzymes, ECG changes, and reduced ejection fraction, which favor acute left ventricular failure. Positive chikungunya IgM antibodies and cardiac MRI findings of mid-wall and subepicardial late gadolinium enhancement in the lateral left ventricular wall supported a diagnosis of chikungunya myocarditis. The absence of significant coronary artery disease on angiography confirmed a non-ischemic etiology.

2D echocardiography helped assess cardiac function and follow-up, while cardiac MRI provided noninvasive confirmation of myocarditis and avoided unnecessary invasive procedures. In resource-limited settings, confirmation of viral etiology can be challenging without PCR, serology, or advanced diagnostics, which may contribute to underreporting of chikungunya myocarditis.

Management of chikungunya myocarditis is primarily supportive. Our patient responded well to diuretics, oxygen, and noninvasive ventilation. He showed significant recovery of his ventricular function on follow-up echocardiography.

This case emphasizes the importance of considering viral myocarditis in patients presenting with acute heart failure or elevated troponin after a febrile illness, especially in endemic regions. Recognizing risk factors can help predict mortality and guide close monitoring. Early diagnosis using imaging and serology helps to reduce unnecessary interventions.

## Conclusions

Chikungunya myocarditis is a rare but potentially serious complication, particularly in patients with comorbidities. A high index of suspicion is essential in patients presenting with fever and cardiac symptoms, especially in endemic regions. Early evaluation with imaging and serology enables accurate diagnosis and prevents unnecessary interventions. With timely recognition and appropriate care, even high-risk patients can recover cardiac function.
